# Cytogenetic abnormalities in multiple myeloma: association with disease characteristics and treatment response

**DOI:** 10.1038/s41408-020-00348-5

**Published:** 2020-08-11

**Authors:** Nadine Abdallah, S. Vincent Rajkumar, Patricia Greipp, Prashant Kapoor, Morie A. Gertz, Angela Dispenzieri, Linda B. Baughn, Martha Q. Lacy, Suzanne R. Hayman, Francis K. Buadi, David Dingli, Ronald S. Go, Yi L. Hwa, Amie Fonder, Miriam Hobbs, Yi Lin, Nelson Leung, Taxiarchis Kourelis, Rahma Warsame, Mustaqeem Siddiqui, John Lust, Robert A. Kyle, Leif Bergsagel, Rhett Ketterling, Shaji K. Kumar

**Affiliations:** 1 Division of Hematology, Mayo Clinic, Rochester, MN USA; 2 Division of Laboratory Genetics, Department of Laboratory Medicine and Pathology, Mayo Clinic, Rochester, MN USA; 3 Division of Nephrology, Department of Internal Medicine, Mayo Clinic, Rochester, MN USA; 4 Division of Hematology, Department of Internal Medicine, Mayo Clinic, Phoenix, AZ USA

**Keywords:** Cytogenetics, Myeloma

## Abstract

Cytogenetic abnormalities are found in most multiple myeloma (MM) patients. Although their prognostic value has been well studied, there are limited data on the association of primary cytogenetic abnormalities with disease characteristics and treatment response. This study was designed to evaluate these associations. This is a retrospective study including 2027 Mayo Clinic patients diagnosed with MM between February 2004 and February 2018 who had cytogenetic testing by FISH at diagnosis. Translocations t(4;14), t(14;16), t(6;14), and t(14;20) were associated with anemia, beta2microglobulin >5.5 µg/ml and ≥50% bone marrow plasma cells; t(4;14) was associated with higher serum monoclonal protein and plasma cell proliferation. Overall response rate to proteasome inhibitor (PI)-based treatment was higher for IgH translocations compared to trisomies (83% vs. 71%, *P* = 0.002), but was higher for trisomies with immunomodulatory drug (IMiD)-based treatment (87% vs. 75%, *P* < 0.001). Time to next treatment was longer with trisomies than IgH translocation with IMiD-based (32.1 vs. 18.4 months, *P* < 0.001) and PI + IMiD-based (44.0 vs. 27.4 months, *P* = 0.003) treatments. Outcomes were superior with PI + IMiD combinations in all groups. Our results show that t(4;14), t(14;16), t(6;14), and t(14;20) are associated with high-risk disease characteristics, and IgH translocations and trisomies may be associated with better responses to PIs and IMiDs, respectively.

## Introduction

Multiple myeloma (MM) is a clonal plasma cell disorder accounting for 1.8% of all malignancies in the US, 18% of hematologic malignancies, and 2% of all cancer-deaths^1^. It is characterized by significant heterogeneity in clinical characteristics, spectrum of genetic abnormalities and treatment outcomes. The use of interphase fluorescence in situ hybridization (FISH), which has greater sensitivity than conventional cytogenetics to detect aberrations given the low proliferative rate of plasma cells, has revealed abnormalities in the majority of patients^2,3^. Translocations involving the immunoglobulin heavy chain gene (IgH) locus and trisomies of odd numbered chromosomes are considered primary cytogenetic abnormalities, occurring at the early premalignant stages and potentially involved in disease pathogenesis^4^. Amongst all prognostic factors described in MM, FISH abnormalities have been found to be the most predictive of outcomes^5^. Translocation t(4;14), t(14;16) and t(14;20) have been associated with poor prognosis, and their presence identifies high-risk (HR) disease. On the other hand, patients with t(11;14), t(6;14) and/or trisomies are considered to have standard-risk (SR) disease^3,6^. In addition to their prognostic value, there is some evidence that cytogenetic abnormalities may confer unique clinical and immunological disease characteristics, which may underlie their prognostic significance^7–9^. Furthermore, poor outcomes associated with HR cytogenetic groups, have led to efforts to identify treatments and combinations with the potential to improve prognosis of patients with these abnormalities. We designed this study to evaluate the association between primary cytogenetic abnormalities and disease characteristics at diagnosis, and to assess whether there are differences between cytogenetic groups in initial treatment response and response durability to different treatments.

## Patients and methods

This is a retrospective study using data from a prospectively maintained database at Mayo Clinic in Rochester, Minnesota, supplemented by review of electronic medical records. The cohort included patients 18 years or older diagnosed with MM from February 2004 to February 2018, who had cytogenetic analysis by FISH performed within 1 year before diagnosis or within 6 months from the start of first-line treatment. Patients with unavailable FISH results and those with testing performed after 6 months from the start of first-line treatment were excluded. All patients authorized use of their medical record data for research. We collected clinical and laboratory data at diagnosis, data on treatment regimens and treatment responses. Staging was performed in accordance with the international staging system (ISS) for MM^10^. The study was approved by the Mayo Clinic Institutional Review Board. Interphase FISH analysis was performed as described previously^11,12^, on unsorted plasma cells from bone marrow samples identified using cytoplasmic immunoglobulin stain. The following probes were used: *RB1*/LAMP1 (Abbott Molecular, Des Plains, IL, USA) for monosomy 13 or 13q deletion, *TP53*/D17Z1 (Abbott Molecular) for *TP53* deletion or monosomy 17, D3Z1/D7Z1/D9Z1/D15Z4 (Abbott Molecular) for trisomy 3, 7, 9 or 15, *TP73*/1q22 (custom probe) for 1q gain, *MYC* (Abbott Molecular) for 8q24.1 rearrangement, *IgH* (custom probe) for 14q32 rearrangements, and probes targeting the individual *IGH* rearrangements t(11;14)(q13;q32) *CCND1/IgH* (Abbott Molecular), t(4;14)(p16.3;q32) *FGFR3*/*IgH* (Abbott Molecular), t(6;14)(p21;q32) *CCND3*/*IgH* (custom probe), t(14;16)(q32;q23) *IgH*/*MAF* (Abbott Molecular), and t(14;20)(q32;q12) *IgH*/*MAFB* (custom probe). The presence of three signals for CCND1 (11q13) in the absence of a t(11;14) rearrangement is interpreted as trisomy 11. Our probe strategy has been designed to detect trisomies 3, 7, 9, 11, and 15, given that the most commonly observed trisomies involve these chromosomes^12^.

### Statistical analysis

We focused the analysis on primary cytogenetic abnormalities, grouping patients into those with an IgH translocation (with or without trisomies) and those with trisomies of at least 1 chromosome in the absence of an IgH translocation. We first compared baseline disease characteristics according to the primary cytogenetic abnormality: t(11;14), t(4;14), t(14;16), t(6;14), t(14;20), unknown IgH translocation/IgH variable region deletion, and trisomies. HR IgH translocations were defined by presence of any of: t(4;14), t(14;16) or t(14;20);^3,6^ SR translocations included patients with any IgH translocation other than HR translocations. Fisher’s exact test was used to study the association with categorical variables and Kruskal–Wallis test was used for continuous variables. We then focused on the impact of treatment approaches in the different cytogenetic subtypes limiting the analysis to patients with available information on first-line and second-line treatments. First and second lines of treatment were defined in accordance with the International Myeloma Working Group (IMWG) consensus criteria^13^. We included patients who received chemotherapy alone and those who underwent transplant post-induction chemotherapy. Patients were grouped into 1 of 4 categories according to the type of first-line induction chemotherapy: (1) PI (Proteasome inhibitor only), (2) IMiD (Immunomodulatory drug only), (3) PI and IMiD combination, and (4) Others. For each treatment category, the overall response rate (ORR), as best response, defined as partial response (PR) or better, and the rate of at least very good partial response (VGPR), were compared between patients with HR IgH translocations, SR IgH translocations, and patients who had trisomies without IgH translocations. Treatment responses were defined in accordance with IMWG MM response criteria^14^. Response rates were compared using the Fisher’s exact test. Then we compared the time to next treatment (TTNT) between the three groups for each treatment category. TTNT was defined as the time of start of first-line treatment to the time of start of second-line treatment. TTNT was estimated using the Kaplan-Meier method and compared between the groups using the Log-Rank test. For all the tests used, two-sided *P* values <0.05 were considered statistically significant. All statistical analyses were performed using the JMP pro software (SAS, Cary, NC).

## Results

### Prevalence of cytogenetic abnormalities

The study included 2027 patients, diagnosed between February 2004 and February 2018, who had a successful FISH analysis. Among these, 120 (6%) had no abnormality detected by FISH with the probes used. Table 1 shows the prevalence of primary cytogenetic abnormalities with the corresponding number of patients who had testing for each probe. An IgH translocation was identified in 46%, and trisomies were found in 57%. Overall, 40% had trisomies without IgH translocation, 30% had an IgH translocation without trisomies, and 16% had both trisomies and IgH translocation. Primary IgH translocations to partner genes *CCND1, CCND3, MAF, MAFB*, and *FGFR3/MMSET* were mutually exclusive. The most frequently observed primary IgH translocation was t(11;14), found in 16% of patients in the absence of trisomies, and in 3% in the presence of trisomies. t(4;14) was found in 6% in the absence of trisomies, and in 3% in the presence of trisomies. t(14;16) was found in 3% in the absence of trisomies, and in 1% in the presence of trisomies. t(6;14) and t(14;20) were each found in ~1% of patients. IgH variable region deletions or translocations involving partners other than the 5 recurrent partners were seen in 4% and 8% in the absence and presence of trisomies, respectively (Supplementary Fig. 1).

**Table 1:** Prevalence of primary cytogenetic abnormalities in multiple myeloma.

Primary abnormalities	Tested *N*	Abnormality *N* (%)
IgH translocation with trisomies	1959	312 (16)
t(11;14)	1962	58 (3)
t(4;14)	1961	60 (3)
t(14;16)	1961	23 (1)
t(6;14)	1962	9 (<1)
t(14;20)	1962	6 (<1)
Unknown partner/del of IgH region	1959	156 (8)
IgH translocation without trisomies	1959	581 (30)
t(11;14)	1962	315 (16)
t(4;14)	1961	117 (6)
t(14;16)	1961	55 (3)
t(6;14)	1962	9 (<1)
t(14;20)	1962	14 (<1)
Unknown partner/del of IgH region	1959	71 (4)
Trisomies without IgH translocation	1959	791 (40)

*del* deletion, *IgH* immunoglobulin heavy chain locus.

### Association with baseline characteristics

Table 2 shows the association between baseline disease and patient characteristics, and primary cytogenetic abnormalities. There was no difference between the groups in the proportion with ECOG performance status ≥2 (*P* = 0.69) or age ≥70 years (*P* = 0.24). There was a male predominance among patients with t(6;14) (89% vs. 61% in the overall cohort). A higher proportion of patients with anemia (hemoglobin < 10 g/dL) was seen among the t(4;14) (39%), t(14;16) (50%), t(6;14) (50%), and t(14;20) (50%) translocations groups, compared to the other groups (P = 0.003). Similarly, a higher proportion of patients in these groups had thrombocytopenia (platelets < 150 × 10^9^/L) (*P* < 0.001), beta2microglobulin (B2M) levels >5.5 µg/ml (*P* = 0.03), and ISS stage III disease (*P* = 0.04). A higher median bone marrow plasma cell percentage (BMPCs) was also seen in these groups (*P* < 0.001). Patients in the t(14;16), t(6;14) and t(14;20) groups had a higher prevalence of renal dysfunction (creatinine ≥ 2 mg/dL) (*P* = 0.01) and higher urine monoclonal protein (*P* = 0.02). The IgA isotype was most prevalent among patients with t(4;14), whereas patients with trisomies without IgH translocations had the highest prevalence of IgG isotype MM. Patients with t(14;20), and patients with trisomies had the highest prevalence of kappa light chain (LC) myeloma, while patients with t(14;16) had the highest prevalence of lambda LC myeloma. A higher proportion of patients had low albumin levels (≤3.5 g/dL) in the t(4;14) group, compared to the other groups (*P* = 0.03). The prevalence of lytic lesions at diagnosis was highest among patients with t(14;20) (85%). Patients with t(4;14) had higher monoclonal protein concentration at diagnosis (median: 3.8 g/dL, *P* < 0.001); 92% of patients in this group had serum M spike level ≥1 g/dL. LC myeloma was more prevalent among patients with t(11;14) (27%) and those with t(6;14) (31%) (*P* < 0.001). Median plasma cell labeling index (PCLI), a marker of plasma cell proliferation, was highest among patients with t(4;14) and t(14;20); 32% and 100% (3/3) of patients had PCLI ≥ 2% in the two groups, respectively. There were no significant differences in lactate dehydrogenase, or calcium levels between the groups.

**Table 2:** Cytogenetic abnormalities and baseline characteristics.

Parameter	All (whole cohort)	t(11;14)	t(4;14)	t(14;16)	t(6;14)	t(14;20)	Unknown IgH trans/del	Trisomies without IgH trans	*P* value
Age (years)									
Median	64 (57–71)	65 (57–70)	64 (55–70)	64 (57–71)	66 (59–74)	56 (52–65)	63 (56–70)	65 (58–71)	**0.04**
≥70 (vs. <70)	558 (28)	104 (26)	43 (24)	20 (26)	6 (33)	2 (10)	64 (28)	233 (30)	0.24
Male	1243 (61)	260 (66)	96 (52)	33 (42)	16 (89)	10 (48)	135 (59)	500 (63)	**<0.001**
ECOG PS									
≥2 (vs. 0–1)	133 (20)	23 (18)	14 (23)	8 (28)	2 (33)	1 (25)	13 (17)	61 (24)	0.69
Hb (g/dL)									
Median	11.0 (9.5–12.5)	11.1 (9.6–12.9)	10.4 (8.8–11.9)	10.0 (8.8–11.6)	10.6 (7.8–11.8)	10.1 (8.3–11.7)	10.9 (9.4–12.2)	11.0 (9.6–12.5)	**<0.001**
<10 (vs. ≥10)	534 (32)	94 (28)	60 (39)	33 (50)	8 (50)	8 (50)	65 (33)	202 (31)	**0.003**
Platelet count (×10^9^/L)									
Median	211 (161–264)	211 (162–266)	188 (140–233)	171 (109–232)	192 (121–220)	163 (63–211)	216 (164–256)	218 (168–269)	**<0.001**
<150 (vs. ≥150)	227 (20)	39 (17)	31 (30)	22 (44)	4 (33)	3 (30)	28 (20)	81 (18)	**<0.001**
WBC ×10^9^/L) (median)	5.3 (4.0–7.0)	5.4 (4.0–7.1)	5.1 (3.9–6.4)	5.4 (3.5–7.6)	4.3 (3.5–7.9)	4.6 (3.7–6.3)	5.2 (4.1–6.4)	5.3 (4.1–6.9)	0.90
Creatinine (mg/dL)									
Median	1.0 (0.9–1.4)	1.1 (0.9–1.5)	1.1 (0.9–1.5)	1.0 (0.8–1.8)	1.4 (1.0–2.6)	1.6 (0.9–4.9)	1.1 (0.9–1.5)	1.0 (0.8–1.3)	**0.003**
≥2 (vs. <2)	245 (15)	52 (16)	24 (16)	14 (22)	5 (31)	7 (41)	29 (15)	77 (12)	**0.01**
LDH (units/L)									
Median	165 (138–201)	169 (137–194)	153 (130–220)	163 (146–224)	178 (147–269)	190 (168–301)	165 (137–200)	162 (136–198)	0.22
>222 vs. (≤222)	193 (17)	30 (14)	23 (23)	14 (25)	4 (33)	2 (25)	20 (14)	68 (15)	0.11
B2M (µg/ml)									
Median	4.0 (2.8–6.9)	3.7 (2.5–6.4)	4.7 (2.8–7.7)	5.5 (3.3–9.9)	4.9 (2.5–11.1)	6.3 (3.5–20.9)	4.1 (2.9–6.3)	3.9 (2.9–6.4)	**0.001**
>3.5 vs. (≤3.5)	804 (57)	149 (52)	77 (58)	40 (70)	7 (50)	10 (83)	95 (61)	324 (58)	0.06
>5.5 vs. (≤5.5)	468 (33)	88 (31)	54 (41)	28 (49)	7 (50)	6 (50)	52 (33)	175 (32)	**0.03**
Albumin (g/dL)									
Median	3.5 (3.2–3.8)	3.6 (3.3–3.8)	3.4 (3.1–3.6)	3.5 (3.2–3.9)	3.6 (3.1–3.7)	3.6 (3.3–4.0)	3.6 (3.3–3.8)	3.6 (3.2–3.8)	**0.01**
≤3.5 (vs. >3.5)	675 (50)	127 (46)	76 (65)	28 (50)	7 (50)	3 (33)	73 (47)	267 (49)	**0.03**
Calcium (mg/dL)									
Median	9.6 (9.1–10.1)	9.6 (9.1–10.2)	9.6 (8.9–10.2)	9.2 (8.8–9.8)	9.5 (9.4–10.2)	9.3 (8.9–9.9)	9.5 (9.1–10.1)	9.6 (9.1–10.1)	0.10
≥11 (vs. <11)	150 (10)	26 (8)	21 (15)	5 (8)	1 (6)	2 (14)	23 (12)	52 (8)	0.27
Lytic lesions	1058 (68)	221 (71)	74 (57)	28 (47)	12 (71)	11 (85)	120 (68)	432 (70)	**<0.001**
% BMPCs									
Median	50 (30–70)	52 (30–75)	59 (35–80)	68 (31–80)	67 (40–90)	60 (40–80)	53 (30–76)	46 (27–70)	**<0.001**
≥50% (vs. <50%)	975 (54)	220 (62)	97 (59)	46 (64)	13 (72)	12 (71)	122 (58)	357 (50)	**<0.001**
Serum M spike (g/dL)									
Median	2.5 (0.7–3.9)	1.3 (0–3.3)	3.8 (2.4–5.0)	2.3 (0.5–3.9)	0.6 (0–2.9)	2.0 (0.5–3.3)	2.2 (0.4–3.9)	2.8 (1.7–3.9)	**<0.001**
≥1 vs. <1	1132 (72)	157 (53)	131 (92)	44 (72)	4 (31)	10 (67)	121 (69)	509 (82)	**<0.001**
LC MM	187 (13)	71 (27)	4 (3)	8 (13)	4 (31)	2(14)	22 (13)	41 (7)	**<0.001**
Urine M spike (g/24 h)	0.05 (0–0.5)	0.03 (0–0.52)	0.08 (0–0.47)	0.21 (0–0.91)	1.06 (0–5.67)	0.64 (0–2.37)	0.06 (0–0.85)	0.03 (0–0.32)	**0.02**
Urine albumin (g/24 h)	0.05 (0.02–0.13)	0.05 (0.02–0.12)	0.06 (0.02–0.17)	0.06 (0.02–0.18)	0.09 (0.03–0.28)	0.17 (0.03–0.26)	0.06 (0.03–0.19)	0.05 (0.02–0.13)	0.33
Ig Isotype									
IgA	350 (25)	52 (20)	60 (48)	19 (31)	2 (15)	4 (29)	55 (33)	122 (22)	**<0.001**
IgG	848 (60)	123 (47)	61 (48)	35 (56)	7 (54)	8 (57)	89 (53)	398 (70)	**<0.001**
Involved LC									
Kappa	919 (64)	162 (60)	71 (56)	28 (45)	10 (67)	11 (79)	98 (60)	403 (70)	
Lambda	507 (36)	110 (40)	56 (44)	34 (55)	5 (33)	3 (21)	66 (40)	173 (30)	**<0.001**
ISS Stage									
I	303 (24)	73 (28)	24 (20)	11 (20)	5 (38)	1 (9)	32 (22)	126 (25)	
II	504 (39)	95 (37)	44 (36)	15 (28)	1 (8)	4 (36)	60 ((42)	213 (41)	
III	469 (37)	89 (35)	54 (44)	28 (52)	7 (54)	6 (55)	52 (36)	175 (34)	
ISS III (vs. 1/2)	469 (37)	89 (35)	54 (44)	28 (52)	7 (54)	6 (55)	52 (36)	175 (34)	**0.04**
PCLI (%)									
Median	0.8 (0.2–1.5)	0.7 (0.2–1.2)	1.2 (0.6–2.6)	0.8 (0.2–1.2)	1.0 (0.2–2.9)	2.0 (2.0–2.6)	0.8 (0.2–1.6)	0.8 (0.2–1.4)	**0.002**
≥2% (vs. <2%)	118 (19)	18 (15)	18 (32)	1 (4)	1 (25)	3 (100)	17 (20)	46 (18)	**0.001**

*B2M* beta2microglobulin, *BMPCs* bone marrow plasma cells, *del* deletion, *trans* translocation, *Hb* hemoglobin, *IgH* immunoglobulin heavy chain, *ISS* international staging system, *LC* light chain, *LDH* lactate dehydrogenase, *MM* multiple myeloma, *PCLI* plasma cell labeling index, *PS* performance status, *WBC* white blood cell.

Bold values indicate statistical significance *P* values < 0.05.

### Treatment response

**Fig. 1:**
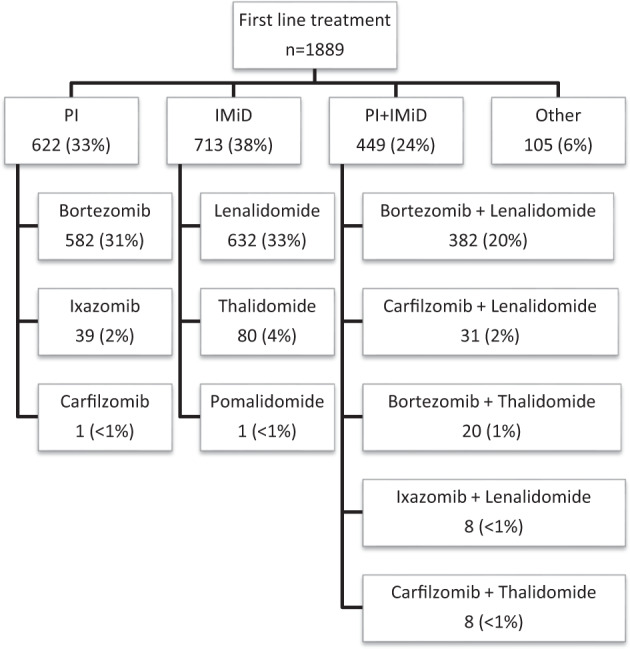
First line treatment. Drugs and combinations used for first-line treatment in patients included in the study. IMiD immunomodulatory drug, PI proteasome inhibitor.

**Fig. 2:**
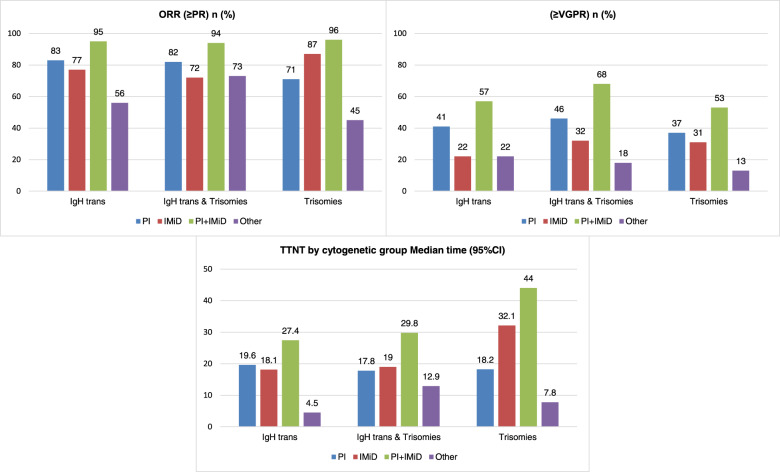
Treatment outcomes for IgH translocation and trisomies groups. Comparison of **a** overall response rate, **b** ≥VGPR rate, and **c** time to next treatment with PI-based (blue), IMiD-based (red), PI + IMiD-based (green) and other (purple) first-line treatments for patients with IgH translocations (without trisomies), trisomies (without IgH translocations) and patients with both IgH translocations and trisomies. IgH immunoglobulin heavy chain, IMiD immunomodulatory drug, PI proteasome inhibitor, trans translocation, VGPR very good partial response.

The median follow up for the entire cohort was 4.3 (interquartile range: 2.3–6.6) years from diagnosis. Treatment data were available for 1889 patients. Among these, 622 (33%) received induction with a PI-based regimen, 713 (38%) received an IMiD-based regimen and 449 (24%) received a PI + IMiD combination (Fig. 1). First-line treatment included autologous stem cell transplantation (ASCT) in 772 (41%) patients and allogeneic stem cell transplantation in 4 (<1%) patients. The responses rates to induction chemotherapy in each cytogenetic group are found in Supplementary table S1. Among patients who received PI-based induction, ORR was higher for those with IgH translocations (83%), compared to those with trisomies without IgH translocation (71%), *P* = 0.002. Conversely, among patients who received an IMiD-based regimen, ORR was higher among patients with trisomies (87%), compared to those with IgH translocations (75%), *P* < 0.001. Among those treated with a PI + IMiD combination, there was no significant difference in ORR between the two groups (94% vs. 96%, *P* = 0.48). There was no significant difference in ORR between patients with HR and SR translocations with PI-based (87% vs. 81%, *P* = 0.19), IMiD-based (81% vs. 73%, *P* = 0.19), PI + IMiD-based (96% vs. 93%, *P* = 0.38) and other treatments. Patients with HR IgH translocations achieved a higher rate of ≥VGPR compared to those with SR IgH translocations when treated with a PI + IMiD based combination (75% vs. 51%, *P* = 0.001). The rates of ≥ VGPR did not differ between the two groups when induction treatment was PI-based (51% vs. 41%, *P* = 0.17) or IMiD-based (23% vs. 26%, *P* = 0.74) (Table 3, Supplementary Fig. S2). Among patients who underwent transplantation post-induction chemotherapy, the ≥VGPR rate to first-line treatment (as best response) was higher in patients with IgH translocations compared to those with trisomies (84% vs. 71%, *P* < 0.001). There was no significant difference in ≥VGPR rate between HR and SR translocations (88% vs. 82%, *P* = 0.21). Figure 2 illustrates the response rates to different induction regimens in patients with IgH translocations, trisomies or both.

**Table 3:** Treatment response by cytogenetic group.

First line therapy	ORR (≥PR) *n* (%)						≥VGPR rate *n* (%)					
	HR trans	SR trans	*P* value	IgH trans (any)	Trisomies	*P* value	HR trans	SR trans	*P* value	IgH trans (any)	Trisomies	*P* value
Overall	214 (88)	428 (80)	**0.006**	642 (83)	557 (83)	1.00	122 (50)	202 (38)	**0.001**	324 (42)	246 (36)	**0.046**
PI	83 (87)	156 (81)	0.19	239 (83)	134 (71)	**0.002**	48 (51)	80 (41)	0.17	128 (44)	69 (37)	0.09
IMiD	52 (81)	136 (73)	0.19	188 (75)	261 (87)	**<0.001**	15 (23)	49 (26)	0.74	64 (26)	92 (31)	0.22
PI + IMiD	73 (96)	124 (93)	0.38	197 (94)	148 (96)	0.48	57 (75)	69 (51)	**0.001**	126 (60)	81 (53)	0.17
Other	6 (75)	12 (57)	0.67	18 (62)	14 (45)	0.21	2 (25)	4 (19)	1.00	6 (21)	4 (13)	0.50

*IgH* immunoglobulin heavy chain, *IMiD* immunomodulatory drug, *PI* proteasome inhibitor, *PR* partial response, *trans* translocation.

Bold values indicate statistical significance *P* values < 0.05.

### Time to next treatment

**Fig. 3:**
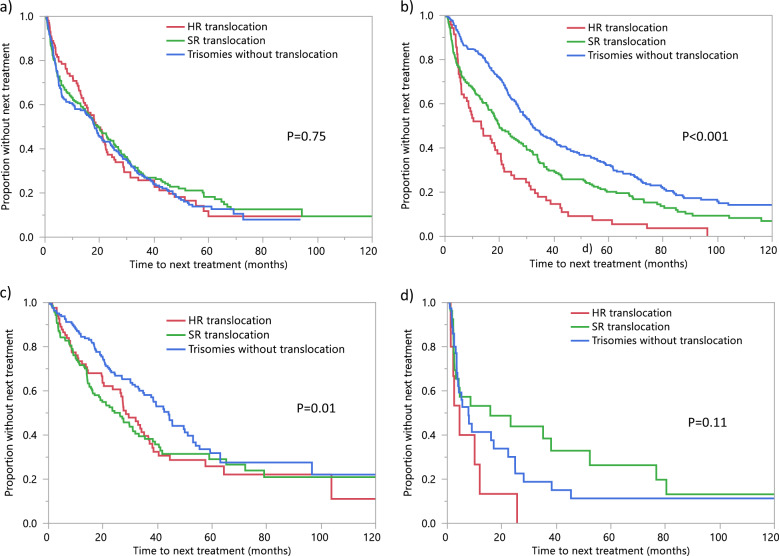
TTNT by cytogenetic group. Comparison of the time to next treatment (TTNT) between patients with high-risk IgH translocations (red curve), those with standard-risk IgH translocations (green curve), and those with trisomies without IgH translocation (blue curve) receiving **a** PI-based, **b** IMiD-based, **c** PI + IMiD-based treatment and **d** other treatments. IgH immunoglobulin heavy chain, IMiD immunomodulatory drug, PI proteasome inhibitor.

The TTNT for the IgH translocation and trisomies groups is found in Supplementary Table S2. For patients treated with a PI-based regimen, there was no significant difference in TTNT between patients with IgH translocation and those with trisomies (19.6 vs. 18.2 months, *P* = 0.48). Similarly, there was no difference in TTNT between patients with HR and SR IgH translocations (19.1 vs. 19.6 months, *P* = 0.76). Among patients treated with an IMiD-based regimen, TTNT was significantly longer in patients with trisomies compared to those with IgH translocation (32.1 vs. 18.4 months, *P* < 0.001). Similarly, TTNT was longer for patients with SR compared to HR translocations (19.8 vs. 13.3 months, *P* = 0.007). Among patients treated with PI + IMiD based regimen, TTNT was also significantly longer for patients with trisomies compared to those with IgH translocations (44.0 vs. 27.4 months, *P* = 0.003). There was no significant difference in TTNT for patients with HR and SR IgH translocations (28.4 vs. 26.2 months, *P* = 0.79) (Table 4). The TTNT curves are presented in Fig. 3. When the analysis was limited to patients who underwent transplantation immediately following frontline induction, there was a trend towards longer TTNT in patients with trisomies compared to IgH translocations for PI-based (36.4 vs. 31.5 months, *P* = 0.72) and IMiD-based (38.7 vs. 31.2 months, *P* = 0.08) treatment. TTNT was significantly longer in patients with trisomies with PI + IMiD-based (45.4 vs. 33.1 months, *P* = 0.04) treatments. When restricting the analysis to patients who did not undergo transplantation post-induction chemotherapy, TTNT was significantly longer for patients with trisomies compared to those with IgH translocations in patients treated with IMiD-based (26.6 vs. 9.9 months, *P* < 0.001), or PI + IMiD-based (38.4 vs. 14.0 months, *P* = 0.02) regimens, and was similar among those treated with a PI-based regimen (5.0 vs. 6.0, *P* = 0.80). There was no significant difference in TTNT between the HR and SR translocation groups for PI- (29.0 vs. 33.0 months, P = 0.51), IMiD-(25.4 vs. 32.4 months, *P* = 0.37) or PI + IMiD-(32.9 vs. 33.1 months, *P* = 0.92) based regimens among patients who underwent transplant post-induction chemotherapy. Among those who received a non-transplant-based approach as first-line treatment, TTNT was longer in those with SR compared to HR translocations with IMiD-based treatment only (12.7 vs. 8.0, *P* = 0.0498); there was no significant difference in TTNT between the 2 groups with PI-based (9.1 vs. 4.6 months, *P* = 0.89) or PI + IMiD-based (14.2 vs. 13.9 months, *P* = 0.82) treatment.

**Table 4:** Time to next treatment by cytogenetic group.

First line induction treatment	TTNT by cytogenetic group Median time (95%CI) (months)					
	HR translocation	SR translocation	*P* value	IgH translocation (any)	Trisomies	*P* value
Overall	19.8 (15.7–22.2)	20.7 (18.0–24.2)	0.19	20.3 (18.3–22.7)	29.1 (26.2–32.0)	**<0.001**
PI	19.1 (14.7–22.4)	19.6 (14.8–25.1)	0.76	19.6 (15.9–22.4)	18.2 (14.7–23.8)	0.48
IMiD	13.3 (7.0–19.2)	19.8 (16.0–26.7)	**0.007**	18.4 (15.3–21.4)	32.1 (28.6–38.7)	**<0.001**
PI + IMiD	28.4 (23.6–36.8)	26.2 (16.9–31.8)	0.79	27.4 (21.2–32.0)	44.0 (35.1–51.1)	**0.003**
Other	4.5 (1.3–11.9)	15.8 (2.7–52.3)	**0.03**	8.5 (2.6–25.6)	7.8 (3.5–22.4)	0.95

*IgH* immunoglobulin, heavy chain, *IMiD* immunomodulatory drug, *PI* proteasome inhibitor, *trans* translocation.

Bold values indicate statistical significance *P* values < 0.05.

## Discussion

Consistent with previous studies, IgH translocations and trisomies were detected in a large subset of patients (46% and 57%, respectively) with newly diagnosed MM^12,15,16^, and IgH translocations were more frequent in non-hyperdiploid myeloma^17^. Among recurrent translocations, t(11;14) has been the most commonly detected, followed by t(4;14) and t(14;16)^5,12^, which is also consistent with our findings. In this study, 6% of patients had no cytogenetic abnormalities detected by FISH. However a subset of these patients had insufficient cells to allow testing using all probes, and thus the prevalence of “normal cytogenetics” in our sample is likely lower; in a previous study by our group, 3% of patients had normal cytogenetics^12^.

Few studies have previously shown that certain primary cytogenetic abnormalities are associated with unique clinical and immunological disease features. t(11;14) translocation has been found to be associated with IgE and IgM heavy chain isotypes^18^, non-secretory MM^7,18^, LC MM^5^, lower serum monoclonal protein levels (<1 g/dL), and lower PCLI^19^. In this study, we found that t(11;14) was associated with lower B2M levels, monoclonal protein concentration and PCLI, LC MM and lower stage disease. However, 62% of patients with t(11;14) had ≥50% BMPCs compared to 54% in the entire cohort. In contrast, t(4;14) translocation has been associated with ISS stage III disease^7^, IgA isotype^7,9^, higher serum monoclonal protein^5^ and B2M > 3^7,9^, which is consistent with our findings. t(4;14) was also associated with non-secretory MM in one study^7^. In a previous study from our group, PCLI was higher among patients with t(14;16), and similar to our findings, t(14;16) was associated with the lambda LC isotype^5^. In another study by Avet-Loiseau et al., there was no association between t(11;14) or t(4;14) and LC isotype or with degree of renal dysfunction^9^. In this study, we found a higher proportion of renal dysfunction (Cr ≥ 2) among patients with t(14;16), t(6;14) and t(14;20) translocations. Greenberg et al. studied the association between cytogenetic subtypes and clinical presentation of end-organ damage. Patients with t(14;16) were more likely to have renal failure as the predominant myeloma-defining event on presentation, whereas t(11;14) and t(6;14) patients were more likely to present with bone disease^8^. There is also evidence that cytogenetic abnormalities confer unique biologic features; t(11;14) was found to be associated with lymphoplasmacytoid morphology, while t(4;14) was associated with immature plasma cell morphology^7^.

In addition to their clinical and biologic significance, we sought to assess if primary cytogenetic abnormalities were associated with differences in response to induction treatment with novel agents. Interestingly, we found that patients with IgH translocation had higher response to PI-based first-line induction treatment compared to patients with trisomies without IgH translocations; however there was no difference in TTNT. Conversely, patients with trisomies had a higher response to an IMiD-based induction regimen and longer TTNT compared to those with IgH translocation. Use of PI-based induction has been associated with improved complete response rates in high-risk cytogenetic groups, specifically with t(4;14). However, these results have not been consistent in all studies^20–22^. In our study, the patients with HR IgH translocations achieved a higher rate of ≥VGPR compared to those with SR IgH translocations when treated with a PI + IMiD based combination. However, the rates of ≥VGPR did not differ between the two groups when the induction treatment was PI-based or IMiD-based. Despite evidence that PI + IMiD based combinations improve the prognosis of patients with high-risk cytogenetics, particularly t(4;14) translocation^23^, there has not been previous evidence of superior responses in patients with high-risk translocations compared to those with standard-risk disease for various PI + IMiD based combinations^24,25^. Variables outcomes with treatment have been reported even within individual cytogenetic groups^26^. This may reflect heterogeneity in patient and disease characteristics within individual cytogenetic groups including the presence of specific secondary cytogenetic abnormalities.

This study is limited by its retrospective nature and small sample sizes for uncommon cytogenetic abnormalities like t(6;14) and t(14;20). In this paper, we did not evaluate the impact of concurrent secondary abnormalities on clinical characteristics and outcomes; the impact of these abnormalities should be addressed in future studies.

In conclusion, cytogenetic abnormalities are associated with unique clinical and immunological characteristics of multiple myeloma at diagnosis. Certain abnormalities may also influence response to various treatments including novel agents; patients with trisomies may benefit from IMiD-based combinations, while patients with IgH translocation may have better responses to PI-based treatment.

Further studies are needed to confirm these findings, which may allow treatment selection in the future to be guided by cytogenetic profile.

## Supplementary information

Supplementary material
