# Supplementary material for: Cytogenetic abnormalities in multiple myeloma: association with disease characteristics and treatment response
Source: Blood Cancer J. 2020 Aug 11;10(8):82. doi: 10.1038/s41408-020-00348-5 (PMC7419564; doi:10.1038/s41408-020-00348-5)
Supplement: Supplementary file 1 — Supplementary material [file 41408_2020_348_MOESM1_ESM.pdf]

Supplementary material:

Supplementary Table S1:

First line induction treatment	ORR (≥PR) by cytogenetic abnormality n (%)							P value
	**t(11;14)**	**t(4;14)**	**t(14;16)**	**t(6;14)**	**t(14;20)**	**Unknown IgH trans/del**	**Trisomies**	
**Overall**	253 (78)	137 (88)	62 (90)	13 (76)	15 (83)	162 (83)	557 (83)	0.11
**PI**	99 (80)	62 (90)	15 (79)	7 (100)	6 (86)	50 (81)	134 (71)	**0.01**
**IMiD**	76 (72)	35 (76)	16 (94)	2 (33)	1 (100)	58 (77)	261 (87)	**<0.001**
**PI+IMiD**	72 (89)	38 (100)	28 (97)	4 (100)	7 (78)	48 (98)	148 (96)	**0.03**
**Other**	6 (50)	2 (67)	3 (75)	-	1 (100)	6 (67)	14 (45)	0.58

Supplementary table S1: **Overall response and primary cytogenetic abnormalities.** Comparison of overall response rate (ORR) to PI-, IMiD- and PI+IMiD- based first-line treatments between patients with different primary cytogenetic abnormalities: t(11;14), t(4;14), t(14;16), t(6;14), t(14;20), unknown IgH trans/del, trisomies without IgH translocation. Abbreviations: *del: deletion*, *IgH: immunoglobulin heavy chain, IMiD: immunomodulatory drug, PI: proteasome inhibitor, trans: translocation.*

Supplementary table S2:

First line induction treatment	TTNT by cytogenetic abnormality median time (95%CI) (months)							P value
	**t(11;14)**	**t(4;14)**	**t(14;16)**	**t(6;14)**	**t(14;20)**	**Unknown IgH trans/del**	**Trisomies**	
**Overall**	19.3 (15.6-23.3)	18.6 (14.1-22.0)	20.3 (11.0-26.4)	25.3 (2.4-NR)	32.0 (8.0-NR)	25.3 (18.3-30.0)	29.1 (26.2-32.0)	**<0.001**
**PI**	19.0 (10.7-25.1)	18.6 (13.3-22.4)	18.0 (7.5-22.6)	40.6 (1.5-NR)	NR (14.7-NR)	18.1 (13.9-33.0)	18.2 (14.7-23.8)	0.06
**IMiD**	19.3 (15.2-24.6)	10.4 (6.0-18.1)	13.3 (4.8-34.1)	11.0 (2.1-NR)	34.1 (25.4-42.8)	26.0 (18.0-31.2)	32.1 (28.6-38.7)	**<0.001**
**PI+IMiD**	19.7 (14.0-29.8)	29.4 (23.6-36.8)	26.7 (11.0-NR)	12.8 (0.86-NR)	19.8 (3.2-38.1)	31.0 (31.1-65.2)	44.0 (35.1-51.1)	**0.02**
**Other**	3.7 (2.2-76.7)	2.4 (1.3-25.6)	10.0 (4.5-11.9)	-	1.3	30.6 (2.3-80.4)	7.8 (3.5-22.4)	**<0.001**

Supplementary table S2: **TTNT and primary cytogenetic abnormalities.** Comparison of the time to next treatment (TTNT) with PI-, IMiD- and PI+IMiD- based first line treatments between patients with primary cytogenetic abnormalities: t(11;14), t(4;14), t(14;16), t(6;14), t(14;20), unknown IgH trans/del, trisomies without IgH translocation. *Abbreviations: del: deletion*, *IgH: immunoglobulin heavy chain, IMiD: immunomodulatory drug, PI: proteasome inhibitor, trans: translocation*

Supplementary figure S1:

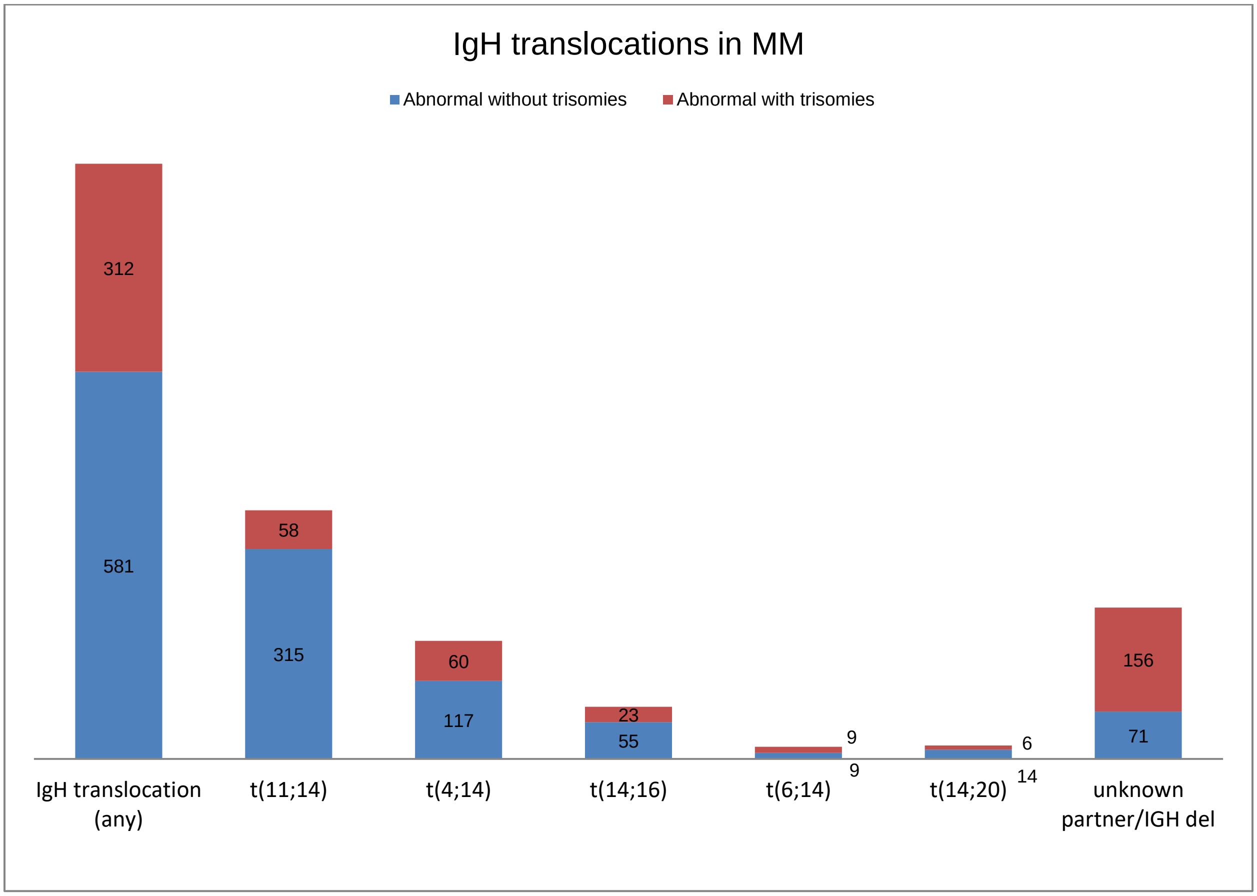

Supplementary figure S1: **IgH translocations in patients with MM.** Number of patients with FISH-detected IgH translocations in the presence of trisomies (red) and IgH translocations in the absence of trisomies (blue). *Abbreviations: del: deletion, IgH: immunoglobulin heavy chain locus, MM: multiple myeloma*

Supplementary figure S2:

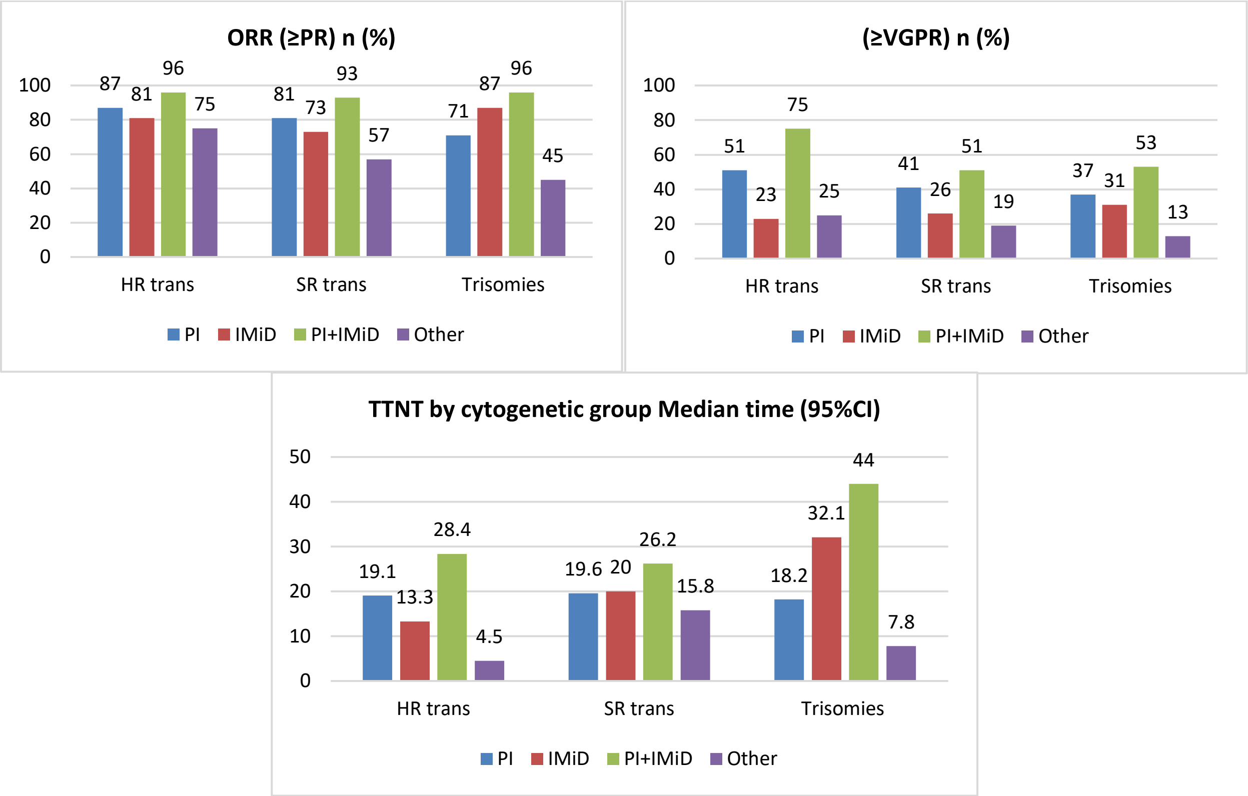

Supplementary figure S2: **Treatment outcomes for HR translocation, SR translocation and trisomies groups.** Comparison of a) overall response rate, b) ≥ VGPR rate and c) time to next treatment with PI-based (blue), IMiD-based (red), PI+IMiD-based (green) and other (purple) first-line treatments for patients with HR IgH translocations, SR IgH translocations, and trisomies. Abbreviations: *HR: high-risk, IgH: immunoglobulin heavy chain, IMiD: immunomodulatory drug, PI: proteasome inhibitor, SR: standard-risk, trans: translocation, VGPR: very good partial response.*
